# Renal Manifestations in VEXAS Syndrome: A Systematic Review of Clinical Features, Pathology, and Outcomes

**DOI:** 10.31138/mjr.280525.apq

**Published:** 2026-03-01

**Authors:** Akhila Arya, Vishnu Mulavini, Rasiya Hashim, Madhumita Rondla, Jia Wei Tan, Dileep Unnikrishnan

**Affiliations:** 1 Yale New Haven Health/Bridgeport Hospital, Bridgeport, CT, USA;; 2 Department of Orthopaedics, Government Medical College Kozhikode, KL, India;; 3 Franciscan Health Olympia Fields, IN, USA;; 4 Department of Internal Medicine, Texas Tech University, El Paso, TX, USA;; 5 Department of Nephrology, Stanford School of Medicine, Stanford, CA, USA

**Keywords:** VEXAS, UBA1, renal disease, kidney

## Abstract

**Objective:**

This review aimed to synthesise current literature on the clinical presentation, renal pathology, treatment, and outcomes of kidney involvement in VEXAS syndrome.

**Methods:**

We conducted a systematic review in accordance with PRISMA guidelines, including all original English-language publications from 2020 to May 14, 2025, that described any form of renal involvement in VEXAS syndrome. Data extracted included patient demographics, clinical presentation, renal biopsy findings, treatment strategies (including dialysis), clinical outcomes, and cause of death.

**Results:**

We included nine studies (five case series, three case reports, and one retrospective cohort), providing a total of 39 individual patient records. The reported frequency of renal involvement in VEXAS syndrome ranged from 3.6% to as high as 39% at 10-year follow-up. All documented cases involved male patients. The most common clinical presentations included acute kidney injury (AKI), chronic kidney disease (CKD), proteinuria, and haematuria. Among biopsy findings, interstitial nephritis was most frequently reported, followed by glomerulonephritis, minimal change disease, acute tubular injury, and IgA nephropathy. Less frequently, membranous nephropathy, peritubular capillaritis, glomerulosclerosis, and light chain cast nephropathy were also observed. Treatment primarily involved glucocorticoids, with steroid-sparing agents added based on the presence of coexisting non-renal manifestations. Renal outcomes were generally favourable, with most patients achieving recovery and only rare instances of dialysis dependence. The contribution of renal involvement to overall mortality remains unclear.

**Conclusion:**

Renal manifestations in VEXAS syndrome are varied but remain underexplored due to the rarity of the disease. Focused studies on kidney involvement are crucial to improve our understanding of the underlying pathophysiology and its clinical implications.

## INTRODUCTION

VEXAS syndrome (Vacuoles, E1 enzyme, X-linked, Autoinflammatory, Somatic) is a recently described, adult-onset autoinflammatory disease caused by somatic mutations in the UBA1 gene, typically affecting haematopoietic stem cells. Renal involvement was not prominently featured in early reports—for example, among 394 reported cases in a 2024 systematic review, kidney manifestations were not listed among the most commonly affected organ systems.^1^ However, emerging evidence from case reports and series indicates that a subset of VEXAS patients develops significant renal manifestations, including acute kidney injury (AKI), chronic kidney disease (CKD), glomerulonephritis, and renal amyloidosis—often in association with high disease activity. The pathophysiology of VEXAS-associated kidney disease is not yet fully understood, but it is hypothesised that the UBA1-mutant myeloid clone directly infiltrates and damages renal tissue, as cytoplasmic vacuoles and UBA1-mutant cells have been identified in affected organs.^2^ Given that renal complications can lead to substantial morbidity, recognising and characterising kidney involvement in VEXAS is crucial for timely and individualised management. This review provides a comprehensive synthesis of the literature since VEXAS was first described, focusing on renal manifestations, biopsy findings, the frequency of kidney involvement, treatments used, and patient outcomes in human studies.

## METHODS

### Protocol and registration

This systematic review was conducted in accordance with the Preferred Reporting Items for Systematic Reviews and Meta-Analyses (PRISMA) guidelines. The study protocol was prospectively registered with the PROSPERO database (Registration number: 1052844). No patients or members of the public were involved in the conception, design, execution, analysis, or dissemination of this research.

### Eligibility criteria

The inclusion criteria were:

1. Diagnosis of VEXAS syndrome by UBA1 mutation testing
2. Primary renal involvement in the form of proteinuria, haematuria, glomerulonephritis or other glomerular pathology, tubular/interstitial pathology, acute kidney injury, chronic kidney disease
3. Original articles like case reports, case series, observational studies, abstracts, letter to editors or commentaries with data on patient presentation

The exclusion criteria were:

1. No clear diagnosis of VEXAS syndrome
2. Cases with no renal involvement
3. Articles with no patient description
4. Articles not available as full text

**Figure 1.**
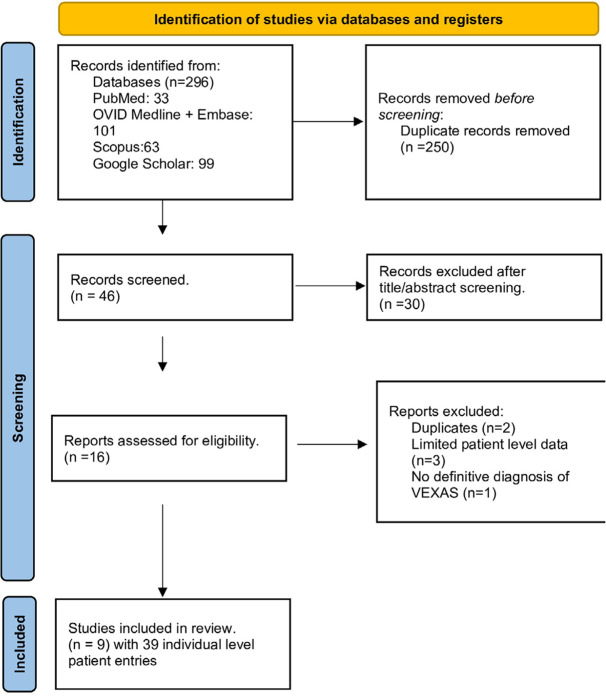
PRISMA-style flow diagram of the search strategy.

The process of article identification, screening, eligibility assessment, and inclusion is depicted in the PRISMA flow diagram (**Figure 1**).

### Information sources

A comprehensive literature search was conducted across PubMed, MEDLINE, Embase, Scopus, and Google Scholar for articles published between 2020 and 2025 that reported renal or kidney involvement in VEXAS syndrome.

### Search strategy

The keywords used were: (“VEXAS syndrome” OR “UBA1”) AND (“renal” OR “kidney” OR “nephritis” OR “glomerulonephritis” OR “AKI” OR “CKD” OR “proteinuria” OR “hematuria” OR “amyloidosis”).

### Selection process

Titles and abstracts of all records retrieved through the search strategy were independently screened by two reviewers (AA and VM) to identify studies that met the predefined inclusion criteria. Full texts of potentially eligible articles were then retrieved and assessed independently by the same reviewers. Discrepancies regarding study eligibility were resolved through discussion. Data extraction was carried out independently by both reviewers, and any differences were resolved by consensus.

### Data collection

A standardised, pre-piloted data extraction form in Google Sheets was used to collect information from the included studies. Key data extracted from each report included clinical and renal presentations, laboratory findings (e.g., proteinuria, haematuria), renal biopsy pathology results, treatments administered specifically for kidney involvement (and overall disease control), and patient outcomes (renal outcomes and overall survival). Any alternative aetiologies for renal injury were also evaluated and documented. Duplicate patient reports across the included studies were removed after detailed manuscript review.

### Quality assessment

Formal tools designed to assess the quality of case reports typically address the following domains: adequacy of exposure ascertainment, adequacy of outcome ascertainment, exclusion of alternative explanations, and reporting of other relevant clinical data. As these elements were already integrated into the article screening and data extraction process, a separate formal risk of bias assessment tool was not employed in this review.

### Data analysis

**Figure 2.**
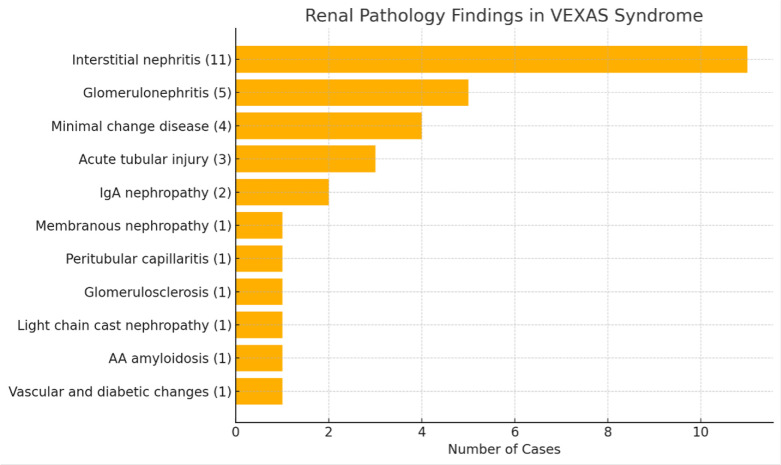
Renal pathology findings in VEXAS Syndrome.

This review was descriptive in nature. Key findings were presented narratively in the text, with detailed case-level data compiled in **Table 1**. Renal biopsy findings were categorised and visually summarised in a figure (**Figure 2**).

**Table 1.** Summary of reported cases of renal involvement in VEXAS Syndrome with kidney biopsy findings.

**First Author (Year)**	**Age/Sex**	**Renal Presentation**	**Kidney Biopsy**	**Treatment**	**Prognosis**
Kalantari et al. (2024)^3^	86/M	AKI	Tubulointerstitial nephritis	Steroids	Alive
Kalantari et al. (2024)	74/M	AKI	Tubulointerstitial nephritis	Steroids + Toci	Alive
Kalantari et al. (2024)	75/M	AKI	Tubulointerstitial nephritis	Steroids	Alive
Kalantari et al. (2024)	76/M	AKI	Tubulointerstitial nephritis	Steroids+ MTX	Alive
Kalantari et al. (2024)	83/M	AKI	Tubulointerstitial nephritis	Steroids	Alive
Kalantari et al. (2024)	78/M	AKI	Light chain cast nephropathy	Steroids + CyBorD + PLEX	Alive
Kalantari et al. (2024)	66/M	AKI	Acute tubular injury, Tubulointerstitial nephritis	Steroids	Alive
Kalantari et al. (2024)	69/M	AKI	Pauci-immune glomerulonephritis	Steroids + Ritux	Alive
Kalantari et al. (2024)	65/M	AKI	.	Steroids	Alive
Kalantari et al. (2024)	78/M	AKI	.	Steroids	Alive
Kalantari et al. (2024)	64/M	AKI	.	Steroids	Alive
Kalantari et al. (2024)	68/M	AKI	.	Steroids	Alive
Kalantari et al. (2024)	71/M	AKI	.	Steroids	Alive
Kalantari et al. (2024)	66/M	AKI	.	Steroids	Alive
Kalantari et al. (2024)	71/M	AKI	.	Steroids	Alive
Kalantari et al. (2024)	57/M	AKI	.	Steroids	Died
Kalantari et al. (2024)	57/M	AKI	.	Steroids	Alive
Kalantari et al. (2024)	68/M	AKI	.	Steroids	Alive
Kalantari et al. (2024)	79/M	AKI	.	Steroids	Died
Kalantari et al. (2024)	59/M	AKI	.	Steroids	Alive
Mathurin et al. (2025)^2^	61/M	CKD, mild Pu	IgA nephropathy	Steroids + Aza	Improved renal function
Mathurin et al. (2025)	72/M	AKI, Pu, Hu	Acute interstitial nephritis	None	Persistent renal impairment; outcome not reported
Mathurin et al. (2025)	69/M	AKI, Pu, Hu	Acute interstitial nephritis	Steroids	Improved renal function
Mathurin et al. (2025)	69/M	Pu, Hu	Acute interstitial nephritis + Minimal change disease	Steroids	Improved renal function
Mathurin et al. (2025)	73/M	AKI, Nephrotic Pu	Pauci-immune glomerulonephritis-Post-infectious glomerulonephritis	Steroids	High creatinine; outcome not detailed
Mathurin et al. (2025)	67/M	Pu	Minimal change disease (with IgA deposits)	Steroids	Improved renal function
Mathurin et al. (2025)	73/M	AKI, Pu	Acute tubular necrosis, mesangial matrix expansion	Steroids	Improved renal function
Mathurin et al. (2025)	77/M	CKD, Pu	Vascular and diabetic	Steroids + Upadacitinib	Stable
Mathurin et al. (2025)	73/M	AKI, Pu	Acute tubular necrosis, mesangial matrix expansion	Steroids + Ruxo	Improved renal function
Mathurin et al. (2025)	59/M	CKD, Nephrotic Pu	AA amyloidosis	Steroids	Not reported
Mathurin et al. (2025)	78/M	AKI, Pu	Acute interstitial nephritis	Steroids	Persistent inflammation; outcome not reported
van der Made et al. (2022)^4^	69/M	Progressive renal insufficiency, Pu, dysmorphic erythrocyturia	Plasma cell-rich interstitial infiltrate (MPO+, CD68+)	Steroids, MTX, Toci, MMF, Cyclophosphamide, Anakinra	Stable
van der Made et al. (2022)	47/M	Pu, microscopic Hu	No evidence of Vasculitis	Steroids, AZT, Anakinra, Ritux, Canakinumab	Stable
Murillo-Chavez (2024)^11^	77/M	Clinical suspicion for ANCA V - rapid rise in Cr, trace Pu	Glomerulosclerosis, tubular injury, arteriolosclerosis	Steroids	Died from pneumonia
Muratore et al. (2022)^8^	75M	Rapidly progressive renal failure	Necrotising and crescentic glomerulonephritis	Steroids, Ritux, MMF, IVIg	NA
Nazario et al. (2025)^12^	71/M	Acute on CKD	C3 Glomerulonephritis	Steroids, Tocilizumab, Siltuximab	Renal function stable
Yildirim et al. (2023)^13^	67/M	AKI, Pu	membranous nephropathy	Steroids, Splenectomy	Renal function improved
Dave et al. (2023)^14^	75/M	AKI	Proliferative crescentic GN	NA	NA
Koster et al. (2021)^15^	71/M	NA	Peritubular capillaritis	RTX, MMF, IFX, TCZ, TOFA, DEC	Alive

AKI: acute kidney injury; Aza: azathioprine; CKD: chronic kidney disease; Cr: creatinine; CyBorD: cyclophosphamide + bortezomib + dexamethasone; DEC: decitabine; Hu: hematuria; GN: Glomerulonephritis; MTX: methotrexate; NA: not available; PLEX: plasma exchange; Pu: proteinuria; Ritux: rituximab; Ruxo: ruxolitinib; Toci: tocilizumab; V: vasculitis.

## RESULTS

We included nine studies (five case series, three case reports, one retrospective cohort study), which together yielded 39 individual-level patient entries (**Table 1**).

### Epidemiology of Renal Involvement in VEXAS Syndrome

The frequency of renal manifestations in VEXAS is not widely reported and was initially thought to be rare. In a retrospective cohort study conducted in the United States, Kalantari et al. reported a cumulative incidence of acute kidney injury (AKI) of 6.2% at 1 year and 39% at 10 years.^3^ A Dutch series of 12 men with VEXAS noted renal involvement in 2 patients (∼17%).^4^ In a Swiss national retrospective cohort study, only 1 out of 17 patients was reported to have AKI.^5^ Similarly, an early report from the United Kingdom by Poulter et al. in 2021 documented renal involvement in 1 of 10 patients.^6^ In a multicentre cross-sectional study conducted in Spain, Escudero et al. reported renal involvement in 8 out of 39 patients (20.5%).^7^ In a French nationwide series, kidney biopsies were performed in 11 out of 303 VEXAS patients (∼3.6%), suggesting that clinically overt renal disease requiring biopsy is relatively uncommon.^2^

### Clinical Presentation of Renal Disease in VEXAS

All reported patients were male, with ages ranging from 47 to 86 years. Clinical presentations varied from insidious CKD with gradual decline in GFR to abrupt-onset AKI. Proteinuria was common; in a French cohort, 5 out of 11 patients had nephrotic-range proteinuria. Haematuria and sterile pyuria were observed in roughly half of the cases. Escudero et al. reported prerenal acute kidney injury (AKI) in two patients, AKI during a disease flare in one patient, proteinuria in two patients, nephrotic syndrome in one patient, chronic kidney disease (CKD) in one patient, and biopsy-proven IgA nephropathy in one patient.^7^ In the cohort described by Madurin et al., only 3 out of 11 patients experienced AKI episodes that were temporally linked to VEXAS flares.^2^ Among the 20 patients in the Mayo retrospective cohort, CRP elevation was significantly associated with time to the first AKI event.^3^ In a retrospective case series from Italy, Muratore et al. described a patient initially diagnosed with MPA based on crescentic glomerulonephritis (GN) in kidney biopsy and positive ANCA-MPO, who was later diagnosed with VEXAS syndrome.^8^

### Histopathologic Findings on Renal Biopsy

Renal biopsy findings, though limited to a few series and reports, reveal heterogeneous histopathology in VEXAS patients. Tubulointerstitial nephritis is a common presentation, with interstitial nephritis often considered a hallmark of VEXAS-related renal pathology.^2^ In the Mayo cohort, six patients underwent kidney biopsy; plasma cell–rich interstitial nephritis was the most common finding, seen in 3 of 6 patients.^3^ In the French biopsy series, nearly all cases demonstrated some degree of inflammatory interstitial infiltrate—often composed of monocytes, neutrophils, and plasma cells—even in patients with a primary glomerular process. Myeloperoxidase and CD68 staining were positive in all, and UBA1 mutations were present in 7/8 samples.^2^ Other glomerular pathologies observed included pauci-immune glomerulonephritis, IgA nephropathy, minimal change disease, membranous nephropathy, peritubular capillaritis, C3 glomerulonephritis and AA amyloidosis. Some biopsies revealed complex pathology with more than one disease process involved, highlighting that VEXAS patients—often older and with comorbidities—may have multiple concurrent renal pathologies.

### Management Strategies for VEXAS-Associated Renal Disease

Glucocorticoids remain the cornerstone of treatment for VEXAS flares, including renal involvement. In the Mayo cohort, all patients showed significant improvement in kidney function and inflammatory markers with steroid therapy.^3^ However, frequent relapses and difficulty with steroid tapering highlight the need for steroid-sparing agents. Conventional immunosuppressants such as cyclophosphamide, methotrexate, azathioprine, and mycophenolate mofetil have been reported. Targeted cytokine therapies have shown promise in refractory VEXAS and have been used in cases with renal involvement. These include tocilizumab (anti-IL6), anakinra (anti-IL1), and JAK inhibitors such as ruxolitinib and tofacitinib. Hypomethylating agents like azacitidine have been used for associated MDS. For renal disease, azacitidine may help indirectly by suppressing the mutant clone and reducing inflammation.^9^ Early reports suggest that hematopoietic stem cell transplantation (HSCT) can induce remission in VEXAS, though it carries substantial risk. Data on renal outcomes post-transplant are limited. In the Mayo Clinic study, two patients underwent HSCT—one achieved normal renal function, while the other had stable CKD due to cardiorenal syndrome.^3^

### Renal and Overall Patient Outcomes

The renal prognosis in VEXAS varies depending on the severity of involvement and response to therapy. In the Mayo cohort, AKI episodes generally improved with high-dose prednisone, with most patients regaining baseline creatinine levels. However, nearly all experienced relapses upon steroid tapering.^3^ In the French series of biopsied patients, renal outcomes were described as “usually favourable” following intensified therapy. Dialysis dependence has not been commonly reported.^2^

Overall, reported mortality rates in VEXAS range from approximately 15% to 50% across different cohorts. In the Mayo Clinic study of 81 patients, 22% died during the follow-up period.^3^ The Dutch series reported around 50% mortality at approximately four years of follow-up, highlighting the serious nature of the disease.^10^

## DISCUSSION

This systematic review summarises the existing evidence on renal involvement in VEXAS syndrome. While early descriptions suggested rarity, multicentre studies now indicate that clinically significant renal disease affects 3–20% of patients, most commonly manifesting as AKI, proteinuria, or haematuria. Biopsy-proven renal involvement appears uncommon in published series. However, this likely underrepresents the true burden of kidney disease in VEXAS, as biopsies were frequently avoided in critically ill patients, particularly those with coagulopathy or thrombocytopenia, and in cases where renal dysfunction improved promptly with empirical therapy.^2–6^ The reliance on clinical judgment in these situations may lead to under-recognition of subclinical or histologically distinct renal lesions. The male predominance observed in renal involvement reflects the X-linked pathogenic basis of VEXAS syndrome, in which UBA1 mutations occur on the X chromosome and are typically pathogenic in hemizygous males. Whether AKI episodes are directly associated with VEXAS flares remains uncertain. While some series report AKI episodes coinciding with flares and improving with immunosuppressive therapy, others show no consistent association. The observation that some AKI events improve rapidly with anti-inflammatory therapy supports a possible pathogenic link in at least a subset of patients.^2, 3^

Histopathology is diverse, with plasma cell–rich interstitial nephritis emerging as a characteristic lesion. In a French series, Myeloperoxidase and CD68 immunostaining were positive in all cases, consistent with the myeloid cell lineages involved in VEXAS syndrome. The detection of UBA1 mutations in renal tissue implicates direct injury from myeloid clones. However, the frequent presence of concurrent glomerular pathologies suggests additional immune-mediated or comorbidity-related mechanisms.^2^

Management of renal manifestations in VEXAS has largely been extrapolated from therapies used for systemic VEXAS and for the specific renal lesions identified. High-dose glucocorticoids provide rapid improvement in kidney function but is limited by relapse and steroid dependence. Steroid-sparing strategies—including immunosuppressants, cytokine inhibitors, JAK inhibitors, and hypomethylating agents—are variably employed, with limited renal-specific outcome data. HSCT offers potential disease modification but carries substantial procedural risk, and its role in preventing renal progression is unproven.

Overall, the renal prognosis in VEXAS appears favourable when diagnosis is prompt and immunosuppression is initiated early, although current evidence is limited to small, retrospective cohorts. Mortality in VEXAS remains high, and the independent contribution of renal disease to overall mortality is uncertain. Nevertheless, complications such as severe AKI, dialysis dependence, and amyloidosis can be life-threatening. Prospective studies are needed to more accurately define prevalence, elucidate pathogenic mechanisms—including the potential role of disease flares—and optimise management strategies for kidney involvement in this syndrome.

## CONCLUSION

This review demonstrates that renal manifestations, while not the most common feature of VEXAS, represent a significant aspect of the disease in a subset of patients. Kidney involvement in VEXAS can take diverse forms—most commonly interstitial nephritis and vasculitic glomerulonephritis, but also immune-complex disease and secondary AA amyloidosis. These renal complications often parallel the activity of the systemic disease. Biopsy findings of plasma cell and neutrophil infiltration in the kidneys, along with UBA1 mutation positivity, suggest direct tissue damage by infiltrating mutant myeloid clones.

The mainstay of treatment for VEXAS flares, including renal involvement, is glucocorticoids. Adjunctive therapies such as cytotoxic agents, anti–IL-1 and anti–IL-6 agents, and JAK inhibitors are frequently added. Renal prognosis following HSCT has been favourable in the two patients with AKI reported. Overall, prognosis based on limited case series and reports is generally good, with mortality and dialysis dependence rarely observed.

This review has several limitations, largely due to its descriptive nature and reliance on case reports. Case reports are prone to publication bias and cannot establish causality, which limits the strength of any conclusions drawn. Additionally, milder forms of renal involvement—such as asymptomatic proteinuria or haematuria—may be underreported, potentially leading to an incomplete picture of the true burden of kidney disease in VEXAS. The quality of included reports varied, and in several cases, important clinical details were missing, making interpretation difficult. However, given the rarity of VEXAS syndrome and its renal manifestations—as well as the limited data currently available—we believe this review provides a valuable starting point for understanding patterns of kidney involvement in this emerging condition. Further studies are needed to clarify why some patients develop renal complications and to help guide future treatment approaches.

## Data Availability

All datasets for this study are included in the manuscript and the supplementary files.
